# Dietary Fructose and Early-Onset Colorectal Cancer: Metabolic, Microbial, and Translational Perspectives

**DOI:** 10.3390/microorganisms14092046

**Published:** 2026-09-14

**Authors:** Sarina Tangyingyong, Hala Abu-Fares, Thiti Susiriwatananont

**Affiliations:** 1 Division of Hematology and Medical Oncology, Mayo Clinic, Jacksonville, FL 32224, USA; sarina.tyy@gmail.com; 2 Department of Immunology, Mayo Clinic, Jacksonville, FL 32224, USA; 3 Division of Medical Oncology, Department of Medicine, Faculty of Medicine, Chulalongkorn University and The King Chulalongkorn Memorial Hospital, Bangkok 10330, Thailand

**Keywords:** early-onset colorectal cancer, fructose, high-fructose corn syrup, fructose metabolism, gut microbiome, tumor microenvironment, sugar-sweetened beverages, metabolic reprogramming

## Abstract

Early-onset colorectal cancer (eoCRC) is increasing globally, raising interest in modifiable dietary exposures that may contribute to its development. High fructose intake, particularly from sugar-sweetened beverages and high-fructose corn syrup, has been associated with colorectal neoplasia and eoCRC in epidemiologic studies. Experimental evidence suggests that fructose may promote colorectal tumorigenesis through enhanced uptake and metabolism, polyol pathway activation, glycolytic and lipogenic reprogramming, hypoxic adaptation, and interactions with the tumor microenvironment. Excess fructose reaching the colon may also impair intestinal barrier integrity and alter gut microbial composition and metabolite production. Preclinical models support a direct tumor-promoting effect of fructose independent of obesity, whereas human mechanistic and microbiome data remain limited. This review summarizes current evidence linking fructose exposure with eoCRC and discusses emerging metabolic, microbial, and translational implications, while highlighting key gaps that must be addressed to establish causality and clinical relevance.

## 1. Introduction

Historically considered a disease of aging, colorectal cancer (CRC) has traditionally been diagnosed after age 50, with median ages at diagnosis in the United States of 67 years in women and 65 years in men [1]. However, global epidemiological patterns have shifted substantially over recent decades, with a marked increase in early-onset colorectal cancer (eoCRC), conventionally defined as CRC diagnosed before the age of 50 years [2]. Between 2013 and 2022, CRC incidence among individuals aged 20–49 years increased by approximately 3% annually, establishing CRC as a leading cancer-related cause of mortality in young adults [3,4].

The factors driving the rising incidence of eoCRC remain incompletely understood. Because hereditary cancer syndromes and established genetic susceptibility account for only a minority of eoCRC cases, increasing attention has focused on environmental and lifestyle exposures, particularly those occurring during childhood, adolescence, and early adulthood. Proposed contributors include sedentary behavior, obesity and metabolic dysfunction, consumption of red and processed meats, ultra-processed foods, smoking, alcohol, and exposure to microbiome-derived carcinogens such as colibactin [5,6]. Collectively, these observations have raised the possibility that changes in diet and metabolism occurring early in life may contribute to colorectal carcinogenesis decades earlier than historically observed.

Among these dietary changes, increased consumption of added sugars, particularly fructose-containing sweeteners, has attracted growing attention. High-fructose corn syrup (HFCS), introduced into the U.S. food supply on a large scale during the 1970s, became widely incorporated into sugar-sweetened beverages (SSBs) and many packaged and processed foods. This transition was accompanied by high levels of added-fructose consumption, particularly among children and adolescents, and occurred alongside increasing rates of obesity and metabolic syndrome [7]. Importantly, emerging experimental evidence suggests that the potential effects of fructose on colorectal carcinogenesis may extend beyond its contribution to excess caloric intake and systemic metabolic dysfunction. When fructose intake exceeds the absorptive and metabolic capacity of the small intestine, fructose can reach the distal gut, where it may alter microbial ecology, intestinal barrier function, microbial metabolite production, and mucosal inflammatory responses. In parallel, colorectal cancer cells can exploit exogenous and endogenously generated fructose to support metabolic reprogramming, proliferation, and survival.

These observations provide a potential biological link between changing dietary patterns and the increasing burden of eoCRC. In this review, we synthesize epidemiological, preclinical, and translational evidence linking dietary fructose and HFCS exposure with colorectal carcinogenesis, with particular emphasis on its potential relevance to eoCRC. We examine intestinal and tumor-specific fructose metabolism, fructose-induced alterations in the gut microbiome and mucosal barrier, interactions with the immune and inflammatory microenvironment, and emerging strategies targeting fructose metabolism and diet. Finally, we highlight critical gaps between mechanistic observations in experimental models and evidence in humans that must be addressed to determine the contribution of fructose exposure to the rising incidence of eoCRC.

## 2. Dietary Fructose: Absorption and Physiologic Metabolism

**Figure 1:**
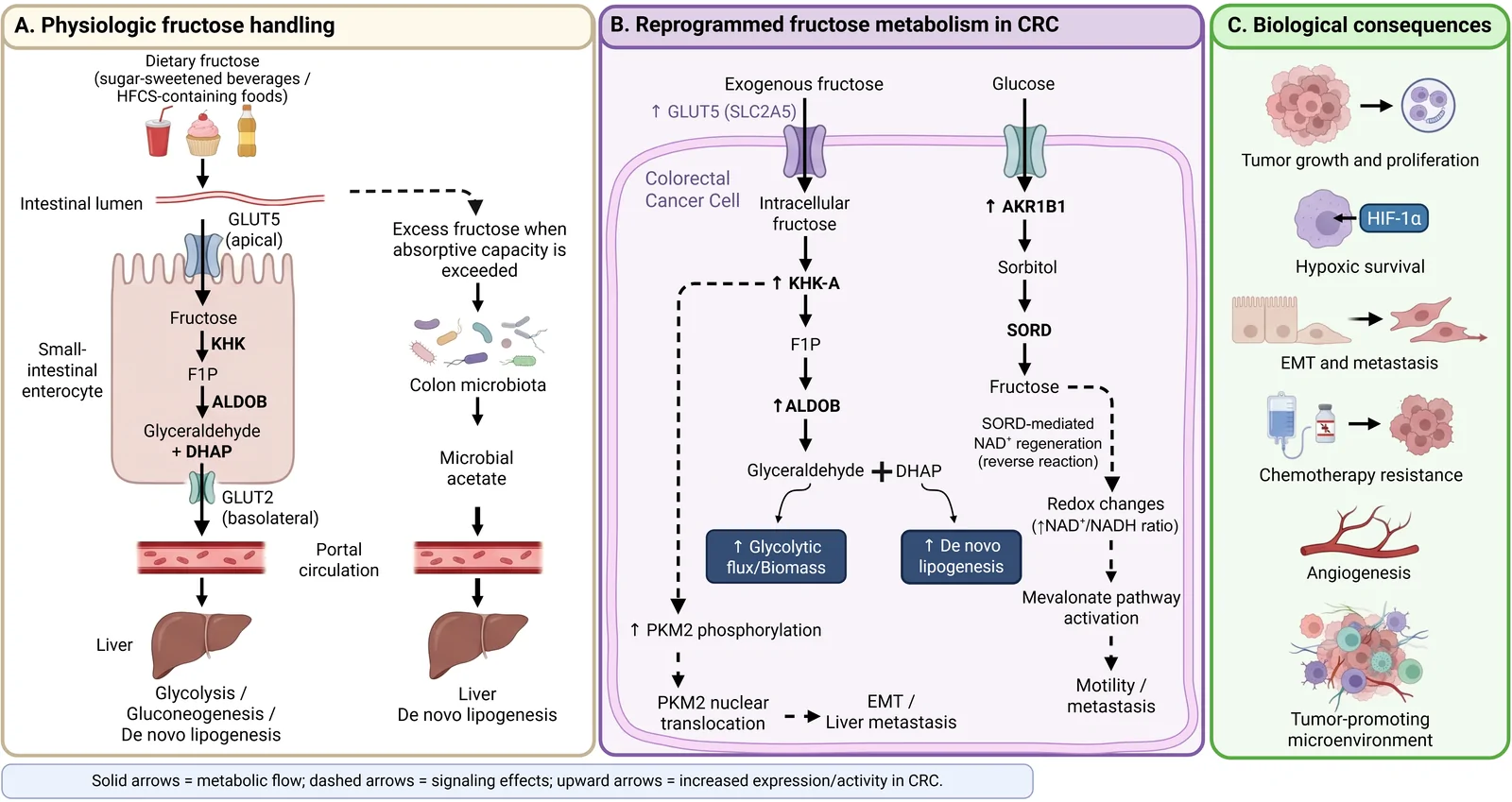
Physiologic fructose handling, metabolic reprogramming, and downstream biological consequences in colorectal cancer: (**A**) Under physiologic conditions, dietary fructose is absorbed by small-intestinal enterocytes primarily through GLUT5 and released into the portal circulation via GLUT2. In enterocytes and hepatocytes, fructose is phosphorylated by ketohexokinase (KHK) to fructose-1-phosphate (F1P), which is subsequently cleaved by aldolase B (ALDOB) into triose intermediates that enter central metabolic pathways. When fructose intake exceeds small-intestinal absorptive capacity, unabsorbed fructose reaches the colon and can be metabolized by the gut microbiota, generating acetate that contributes to hepatic de novo lipogenesis. (**B**) In colorectal cancer (CRC), fructose metabolism is reprogrammed through increased fructose uptake and altered expression or activity of key metabolic enzymes, including GLUT5, KHK-A, ALDOB, AKR1B1, and SORD. CRC cells can utilize both exogenous fructose and endogenously generated fructose via the polyol pathway. (**C**) These alterations support tumor progression by promoting glycolytic and lipogenic reprogramming, hypoxic adaptation, epithelial–mesenchymal transition, angiogenesis, metastatic behavior, and tumor-promoting microenvironmental interactions.

To distinguish normal fructose handling from tumor-associated metabolic reprogramming, this section first summarizes the dietary sources, intestinal absorption, intracellular metabolism, and endogenous production of fructose under physiologic conditions. Section 4 then discusses how these pathways are altered or exploited in CRC cells. These processes are summarized schematically in Figure 1.

### 2.1. Dietary Sources and Intestinal Absorption

The terms fructose, sucrose, high-fructose corn syrup (HFCS), and sugar-sweetened beverages (SSBs) are used distinctly throughout this review. Fructose refers to the monosaccharide itself, whereas sucrose is a disaccharide composed of approximately equal amounts of glucose and fructose. HFCS is a mixture of free glucose and fructose, with commonly used formulations containing approximately 42% or 55% fructose. SSBs refer to beverages containing added caloric sweeteners and may contain HFCS, sucrose, or other sugars.

HFCS is widely used as a caloric sweetener in SSBs and processed foods, including baked goods, desserts, grain products, and condiments [8]. U.S. dietary survey data from 1977 to 2004 estimated a mean daily fructose intake of approximately 49 g, corresponding to approximately 10% of total energy intake, with the highest consumption observed among adolescents and young adults aged 15–22 years (55–75 g/day). Nonalcoholic beverages and grain products accounted for 46.0% and 17.3% of dietary fructose intake, respectively [9].

Following ingestion, fructose is transported across the apical membrane of small-intestinal enterocytes primarily by glucose transporter 5 (GLUT5; SLC2A5) and subsequently released across the basolateral membrane into the portal circulation, in part through GLUT2 (SLC2A2). The small intestine plays an important role in first-pass fructose metabolism; however, its capacity is limited and depends on the dose and rate of fructose ingestion. Absorbed fructose and its intestinally derived metabolites enter the portal circulation and can subsequently undergo hepatic metabolism, whereas fructose that exceeds small-intestinal absorptive capacity reaches the colon, where it interacts with the gut microbiota [10,11].

### 2.2. Influence of Food Source and Energy Context

The metabolic effects of fructose-containing sugars depend on both food source and energy balance. In a meta-analysis of 169 controlled trials, excess energy from fructose-containing sugars, particularly SSBs, increased body weight, whereas no overall effect was observed in energy-matched substitution trials [12]. A second meta-analysis of 155 controlled study comparisons similarly found no overall harmful effect on glycemic outcomes in energy-matched substitution or subtraction studies, while adverse effects on fasting insulin were observed when sugars provided excess energy or when energy intake was uncontrolled [13]. In both analyses, effects varied by food source, with whole fruit generally showing no adverse effects, whereas SSBs were more often associated with less favorable outcomes. These findings suggest that the effects of fructose-containing sugars should be interpreted within the context of food source and energy balance rather than fructose exposure alone.

### 2.3. Intracellular Fructose Metabolism

Within enterocytes and hepatocytes, fructose is phosphorylated by ketohexokinase (KHK) to fructose-1-phosphate (F1P), which aldolase B (ALDOB) subsequently cleaves into glycolytic trioses (glyceraldehyde and dihydroxyacetone phosphate). These triose intermediates enter central carbon metabolism and can contribute to glycolysis, gluconeogenesis, and de novo lipogenesis [14].

### 2.4. Endogenous Fructose Production and the Polyol Pathway

In addition to exogenous dietary fructose, fructose can be generated endogenously from glucose through the polyol pathway. In this pathway, aldose reductase (AKR1B1) converts glucose to sorbitol, which is subsequently oxidized to fructose by sorbitol dehydrogenase (SORD) [15]. Under normoglycemic conditions, however, flux through the polyol pathway is relatively low, accounting for less than 3% of cellular glucose utilization [16].

### 2.5. Metabolic Consequences of Fructose Handling and the Gut–Liver Axis

Fructose metabolism differs fundamentally from glucose metabolism in several respects. Whereas glucose utilization is widely distributed across tissues and tightly integrated with insulin-dependent metabolic regulation, fructose is predominantly cleared by the intestine and liver and is metabolized largely independently of insulin. Importantly, fructose metabolism generates triose intermediates that enter glycolytic pathways downstream of phosphofructokinase-1 (PFK-1), a major regulatory checkpoint of glycolysis. Consequently, fructose metabolism is less constrained by PFK-1-mediated feedback regulation and can provide substrates for de novo lipogenesis (DNL), including fatty acids and phospholipids [17]. The gut microbiome provides an additional metabolic route for fructose that escapes small-intestinal absorption. Microbial fermentation of fructose can generate acetate, which enters the portal circulation and serves as a substrate for hepatic acetyl-CoA production and DNL, thereby linking dietary fructose exposure to metabolic changes across the gut–liver axis [18] (Figure 1A).

## 3. Dietary Fructose, Sugar-Sweetened Beverages, and Early-Onset Colorectal Cancer

SSBs, a major source of added fructose and HFCS, have been associated with colorectal neoplasia in epidemiologic studies [19]. Of particular relevance to eoCRC, prospective cohort studies suggest that fructose and SSB exposure during adolescence and early adulthood may be associated with later colorectal neoplasia.

In a prospective analysis of 33,106 participants from the Nurses’ Health Study II, each 5% increment in total caloric intake from fructose during adolescence was associated with a 17% higher risk of developing high-risk colorectal adenomas [20]. In a separate cohort of 95,464 women, consumption of two or more servings of SSBs per day in adulthood was associated with approximately a 2.2-fold higher risk of eoCRC compared with consumption of one or fewer servings per week. Each additional daily serving of SSBs during adolescence was associated with a 32% higher risk of eoCRC [21]. These findings support the hypothesis that dietary exposures occurring early in life may contribute to colorectal carcinogenesis before the age of 50 years.

Emerging data also suggest that the association between dietary sugar intake and CRC may vary according to anatomic subsite. Although eoCRC frequently presents in the left-sided (descending colon to rectum), higher consumption of SSBs and total fructose has been associated in some studies with increased incidence and mortality of right-sided (cecum to transverse colon) colon cancers, particularly tumors arising in the cecum [22,23]. One possible explanation is that unabsorbed fructose reaching the proximal colon may create a local metabolic and microbial environment that differs from that of the distal colorectum, although this mechanism has not been established in humans [24].

Supporting the possibility of site-specific biological effects, epigenomic profiling of normal colonic mucosa in African American adults identified substantially more fructose-associated differentially methylated regions in the right colon than in the left colon. In the same study, right-colon organoid models demonstrated fructose-responsive changes in pathways related to glycolysis, lipid metabolism, and cell proliferation [25]. These findings suggest that fructose exposure may exert region-specific effects on the colonic mucosa; however, their relationship to CRC initiation, eoCRC risk, and observed racial disparities remains uncertain.

Overall, epidemiologic studies provide suggestive evidence linking high SSB and fructose exposure, particularly during adolescence and young adulthood, with colorectal neoplasia and eoCRC. However, these findings are observational and remain susceptible to residual confounding by obesity, physical activity, overall dietary quality, total energy intake, and other lifestyle factors. In addition, SSB exposure cannot be considered equivalent to fructose or HFCS exposure, because SSBs contain multiple dietary components and most epidemiologic studies do not isolate fructose-specific effects. Similarly, associations with total sugar intake do not establish that fructose is the causal component. Therefore, current epidemiologic evidence supports an association but does not establish a direct causal relationship between dietary fructose or HFCS and eoCRC.

## 4. Altered Fructose Metabolism in Colorectal Cancer

### 4.1. Exogenous and Endogenous Fructose Utilization by CRC Cells

Against this physiologic background, CRC cells can reprogram fructose transport and metabolism to support proliferation and adaptation to nutrient and oxygen stress. Malignant cells undergo extensive metabolic reprogramming to sustain proliferation and survive under conditions of fluctuating nutrient availability, hypoxia, and oxidative stress. Aerobic glycolysis, commonly referred to as the Warburg effect, allows cancer cells to generate ATP while maintaining a supply of biosynthetic intermediates required for rapid growth [26]. Although glucose remains a major metabolic substrate, several cancer types can also utilize fructose to support proliferation and metabolic adaptation, particularly when canonical glucose-dependent pathways are constrained [27,28].

In addition to utilizing exogenous fructose, malignant cells may generate fructose endogenously through increased flux through the AKR1B1–SORD polyol pathway described above. Under metabolic or hypoxic stress, increased polyol-pathway activity may provide an alternative source of fructose-derived carbon for glycolytic and biosynthetic pathways [29,30]. Because fructose metabolism enters glycolysis downstream of PFK-1, increased fructose utilization may facilitate metabolic flexibility under conditions in which glucose metabolism is limited or tightly regulated [31].

Emerging translational evidence suggests that CRC cells may exploit both exogenous fructose and endogenously generated fructose. In a recent study of human CRC, preoperative oral glucose administration increased circulating fructose and lactate concentrations and reduced the intratumoral glucose-to-fructose ratio, providing evidence consistent with active glucose-to-fructose conversion through the polyol pathway in CRC tissue [32].

### 4.2. Altered Fructose Transport and Enzymatic Pathways in CRC

Several components of fructose transport and metabolism are dysregulated in CRC. The fructose transporter GLUT5, encoded by SLC2A5, is upregulated in colorectal cancer cells and cancer-associated fibroblasts, facilitating fructose uptake and utilization. GLUT5 upregulation has been associated with enhanced tumor-cell proliferation, chemotherapy resistance, and metastatic behavior [33,34,35,36]. Downstream of fructose uptake, ketohexokinase (KHK) phosphorylates fructose to F1P. In CRC, the KHK-A isoform has also been implicated in non-canonical signaling through phosphorylation and nuclear translocation of pyruvate kinase M2 (PKM2), promoting epithelial–mesenchymal transition (EMT) and liver metastasis [37].

ALDOB, which cleaves F1P into triose intermediates, represents another important component of fructose metabolism in CRC. Increased ALDOB expression has been associated with enhanced glycolytic flux, metabolic adaptation to hypoxic and desmoplastic microenvironments, and metastatic colonization [38,39]. In parallel, activation of the polyol pathway through AKR1B1 and SORD provides an endogenous source of fructose and alters cellular redox balance. AKR1B1 overexpression has been linked to oxidative stress, mitogenic signaling, EMT, and M2 tumor-associated macrophage (TAM) polarization, whereas SORD-dependent fructose–sorbitol cycling may promote activation of the mevalonate pathway and metastatic motility [40,41,42,43].

### 4.3. Functional Consequences of Fructose Metabolic Reprogramming

Fructose metabolism also interacts with broader metabolic and signaling networks within the tumor microenvironment. Under hypoxic conditions, fructose can sustain glycolytic flux and protect CRC cells from RIP-dependent necroptosis [44]. High-fructose exposure has additionally been associated with increased hexokinase 2 (HK2) activity and impaired M1 TAM polarization, thereby linking tumor-cell metabolism with immune regulation [45,46]. Furthermore, fructose has been shown to enhance reactive oxygen species (ROS) generation and activate Akt/Src–VEGF signaling, promoting angiogenesis and increased microvascular density in experimental CRC models [47].

Collectively, these findings suggest that altered fructose metabolism in CRC extends beyond simple nutrient utilization. Fructose can contribute to tumor growth through multiple interconnected mechanisms, including metabolic reprogramming, adaptation to hypoxia, EMT (Figure 1B,C, and Table 1), stromal interactions, immune modulation, and angiogenesis. However, the relative importance of these pathways in human CRC, and particularly in early-onset disease, remains incompletely defined.

## 5. Dietary Fructose and Colorectal Tumorigenesis in Preclinical Models

Although SSBs and excessive fructose intake are associated with obesity and metabolic syndrome, both of which are established risk factors for CRC, preclinical studies suggest that fructose may also promote intestinal tumorigenesis through direct local and cell-intrinsic mechanisms that are at least partly independent of systemic metabolic dysfunction.

In wild-type mice, dietary fructose has been shown to enhance enterocyte survival under hypoxic conditions and promote elongation of small-intestinal villi. Mechanistically, F1P inhibits PKM2, thereby reducing oxidative stress and enhancing hypoxia-inducible factor-1 alpha (HIF-1α)-dependent survival signaling. The resulting increase in villus length expands the intestinal absorptive surface area, which can increase nutrient uptake and accelerate adiposity in the context of a high-fat diet. Genetic deletion of *Khk* or pharmacological activation of PKM2 prevented fructose-induced villus elongation and attenuated the associated increase in nutrient absorption, supporting a causal role for KHK-dependent fructose metabolism in this adaptive response [48].

More direct evidence linking fructose to intestinal tumor growth comes from *Apc*-mutant mouse models of colorectal neoplasia. In these models, administration of moderate amounts of HFCS increased both tumor size and histological grade without causing obesity or metabolic syndrome. Neoplastic intestinal epithelial cells actively took up luminal glucose and fructose, and KHK-mediated conversion of fructose to F1P enhanced glycolytic flux and de novo fatty acid synthesis, thereby supplying the energy and biosynthetic substrates required for tumor growth. Importantly, genetic ablation of either *Khk* or fatty acid synthase (*Fasn*) abolished the tumor-promoting effect of HFCS, providing evidence that local sugar metabolism can directly accelerate intestinal tumorigenesis independently of systemic weight gain [49].

Together, these studies demonstrate that fructose can influence intestinal biology at multiple levels, including epithelial adaptation, nutrient absorption, and tumor-cell metabolism. The *Apc*-mutant model provides mechanistic evidence that HFCS can accelerate intestinal tumor growth through KHK-dependent fructose metabolism and lipid synthesis.

## 6. Fructose-Induced Alterations in the Intestinal Barrier and Gut Microbiome

The gut microbiota plays an important role in maintaining intestinal homeostasis through regulation of mucosal integrity, host metabolism, and immune function. Disruption of microbial community structure and function can promote chronic inflammation and colorectal carcinogenesis through multiple mechanisms, including epithelial barrier impairment, altered microbial metabolite production, genotoxicity, and activation of oncogenic signaling pathways [50,51]. Excessive fructose exposure may contribute to this process by increasing the amount of unabsorbed fructose reaching the colon, thereby altering both the intestinal microenvironment and microbial metabolism.

### 6.1. Intestinal Barrier Dysfunction

Preclinical studies demonstrate that high-fructose exposure can impair colonic barrier integrity across several structural levels. At the luminal surface, high dietary fructose reduces mucus thickness and decreases expression of mucin-2 (MUC2), weakening the protective mucus layer [52]. Histologic studies have additionally reported mucosal thinning, epithelial cell shedding, crypt and gland loss, and lamina propria edema following chronic fructose exposure [53,54,55].

Fructose exposure has also been associated with reduced expression of key tight-junction proteins, including zonula occludens-1 (ZO-1), occludin, and claudin-1, together with ultrastructural disruption of intercellular junctions and increased intestinal permeability [53,55,56]. In one experimental model, high-fructose feeding promoted localization of *Akkermansia muciniphila* in the inner colonic mucus layer, where increased mucin degradation was associated with disruption of the epithelial barrier and closer contact between luminal microorganisms and the colonic epithelium [52].

### 6.2. Microbial Dysbiosis

High-fructose diets also alter gut microbial composition in animal models. Reported changes include reduced microbial diversity and shifts in the relative abundance of major bacterial groups, although the direction and magnitude of these changes vary across experimental models [54,55,56,57,58]. Several studies have reported depletion of commensal taxa associated with short-chain fatty acid production, including *Lactobacillus*, *Bifidobacterium*, *Butyricicoccus*, and members of the Muribaculaceae family [52,55,59,60]. In contrast, fructose exposure has been associated with expansion of potentially pro-inflammatory taxa, including *Desulfovibrio*, *Helicobacteraceae*, *Mucispirillum*, and *Tyzzerella* [53,56,57,61,62].

### 6.3. Altered Microbial Metabolites and Inflammatory Signaling

Fructose-induced dysbiosis can alter microbial metabolic output. Several preclinical studies have reported reduced concentrations of protective short-chain fatty acids (SCFAs), including acetate, propionate, and butyrate, following chronic fructose exposure [53,54,60]. Reduced SCFA availability may impair epithelial barrier maintenance and mucosal immune regulation. Fructose exposure has also been associated with altered microbial bile-acid metabolism, including reduced bacterial bile salt hydrolase activity and accumulation of secondary bile acids such as lithocholic acid [45,52].

When barrier integrity is compromised, bacterial products such as lipopolysaccharide (LPS) may translocate across the intestinal mucosa and contribute to local or systemic inflammatory signaling. In animal models, high-fructose diets have been associated with increased endotoxin exposure and metabolic inflammation [60]. Together, these changes provide a potential mechanistic link between excessive fructose intake, microbial dysfunction, impaired epithelial integrity, and a pro-inflammatory intestinal microenvironment.

### 6.4. Evidence from Human Studies

Human data remain limited and are less consistent than findings from preclinical models. Available clinical studies suggest that the effects of dietary fructose on the gut microbiome depend on dose, food matrix, and dietary context (Table 2).

In one pilot dietary intervention, consumption of approximately 100 g/day of fructose within a fruit- and vegetable-rich dietary pattern was associated with enrichment of bacterial groups linked to butyrate production, whereas a comparable amount of refined high-fructose syrup was associated with depletion of several butyrate-producing taxa and a shift toward a less favorable microbial profile [10]. These findings suggest that fructose delivered within whole foods may have different effects from refined fructose-containing sweeteners because of accompanying fiber, phytochemicals, and other dietary components.

In contrast, a short-term randomized crossover study in participants with obesity found that 75 g/day of pure fructose reduced the relative abundance of *Bifidobacterium*, but a similar reduction occurred during an isocaloric glucose intervention, suggesting that this effect may not be fructose-specific. Furthermore, fructose exposure did not significantly alter overall microbial diversity, intestinal permeability, markers of endotoxemia, or gastrointestinal inflammatory biomarkers during the study period [63].

**Figure 2:**
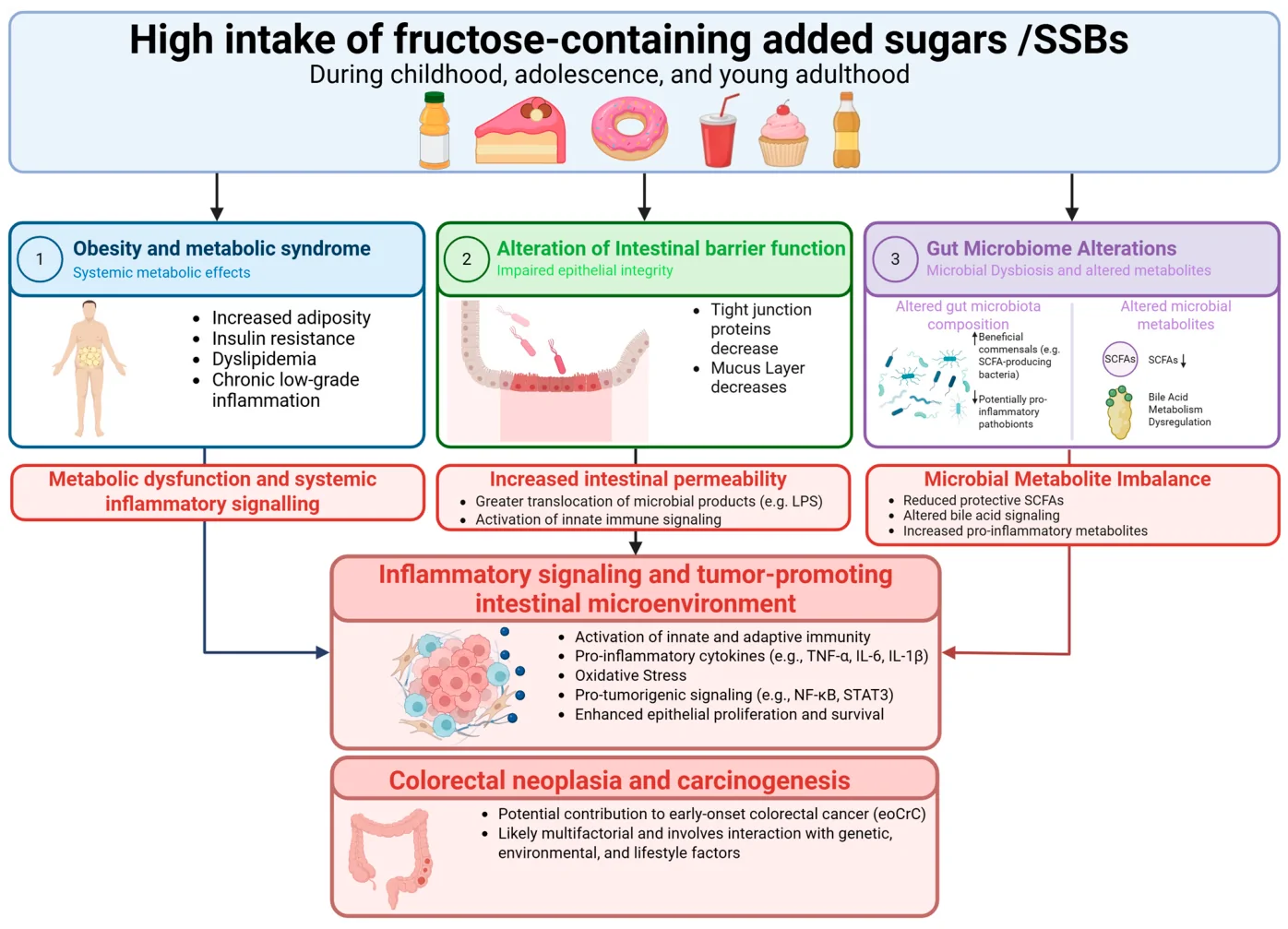
Proposed systemic, intestinal, and microbial pathways linking high intake of fructose-containing added sugars with colorectal carcinogenesis and potential relevance to early-onset colorectal cancer. ↑ = increased relative abundance; ↓ = decreased relative abundance.

Overall, current evidence supports fructose-induced disruption of the intestinal barrier and microbiome in experimental models, whereas corresponding effects in humans remain less clearly established. Interpretation of human studies is complicated by substantial variability in habitual diet, difficulty in accurately quantifying fructose exposure, differences in food matrix, duration of exposure, absorptive capacity, and considerable interindividual variation in baseline microbiome composition. These factors may partly explain the inconsistent findings across studies. The proposed systemic, intestinal barrier, and microbiome-mediated pathways linking excessive fructose exposure with colorectal carcinogenesis and their potential relevance to eoCRC are summarized in Figure 2. Whether fructose-associated microbiome alterations directly contribute to colorectal carcinogenesis or eoCRC risk in humans remains unresolved.

Long-term controlled dietary studies using CRC incidence as an endpoint are unlikely to be feasible because of the prolonged latency of colorectal carcinogenesis. More practical approaches may include observational studies integrating detailed dietary assessment with stool microbiome profiling and colorectal mucosal sampling in individuals undergoing colonoscopy, allowing associations between dietary fructose exposure, microbial features, and colorectal adenomas, other precursor lesions, or tumor characteristics to be examined. However, such studies would remain observational and would not establish causality.

## 7. Therapeutic Perspectives and Translational Opportunities

Although no clinical trial has established fructose-targeted therapy as an effective treatment for CRC, preclinical studies have identified several fructose-related metabolic pathways that may represent experimental therapeutic targets. Some agents targeting these pathways have entered clinical development or are approved for other indications, demonstrating pharmacologic feasibility in humans rather than established efficacy in CRC. Their relevance to CRC, and particularly to eoCRC, remains uncertain.

### 7.1. Targetable Pathways in Fructose Metabolism

#### 7.1.1. Ketohexokinase Inhibition

Ketohexokinase (KHK) is a key enzyme in fructose metabolism and catalyzes the conversion of F1P. In *Apc*-mutant mouse models, genetic disruption of KHK abolished HFCS-associated acceleration of intestinal tumor growth, supporting KHK as a potential therapeutic target [49]. Small-molecule KHK inhibitors, including PF-06835919, have undergone Phase II evaluation in metabolic dysfunction-associated steatotic liver disease and related metabolic disorders, demonstrating that systemic KHK inhibition is pharmacologically feasible in humans [64,65].

#### 7.1.2. Pyruvate Kinase M2 Activation

Fructose-derived F1P can inhibit pyruvate kinase M2 (PKM2), promoting metabolic adaptation under hypoxic conditions. In preclinical models, pharmacological activation of PKM2 with TEPP-46 counteracted F1P-mediated inhibition and attenuated fructose-associated intestinal phenotypes, including enhanced nutrient absorption and tumor-promoting effects [48]. Pharmacologic activation of pyruvate kinase is clinically feasible, as demonstrated by the approval of mitapivat for hereditary hemolytic anemia. In oncology, the oral PKM2 activator TP-1454 has entered early-phase clinical evaluation in patients with advanced solid tumors (NCT04328740).

#### 7.1.3. Aldose Reductase Inhibition

Aldose reductase (AKR1B1) catalyzes the first step of the polyol pathway, converting glucose to sorbitol and thereby contributing to endogenous fructose generation. Preclinical studies implicate AKR1B1 in CRC-associated oxidative stress, proliferative signaling, epithelial–mesenchymal transition, and tumor-associated macrophage polarization [40,41,42]. Epalrestat, an oral AKR1B1 inhibitor approved in several Asian countries for diabetic neuropathy, is currently being investigated in other neoplastic settings, including metastatic triple-negative breast cancer (NCT03244358).

Collectively, KHK, PKM2, and AKR1B1 represent pharmacologically targetable components of fructose-related metabolism with supportive preclinical evidence in CRC and varying degrees of clinical experience in other diseases. However, none of these strategies has yet been validated as a fructose-targeted therapy in patients with CRC. Their clinical relevance, optimal patient selection, and potential efficacy in eoCRC therefore remain to be determined.

### 7.2. Dietary and Microbiome-Directed Interventions

Dietary approaches may provide an additional strategy to reduce the biological consequences of excessive fructose exposure. Soluble dietary fibers can increase proximal intestinal and microbial fructose utilization, thereby reducing the amount of fructose reaching the distal gut. In preclinical models, fiber-adapted microbiota increased fructose clearance and attenuated downstream metabolic consequences, including hepatic lipid accumulation [66].

The food matrix may also influence the effects of dietary fructose. A human intervention study suggests that fructose consumed within fruit- and vegetable-rich dietary patterns may produce different microbiome responses from equivalent amounts of refined high-fructose syrup [10]. These findings support the concept that reducing refined added sugars while maintaining whole-food sources of fructose may be more relevant than indiscriminate fructose restriction.

Microbiome-directed strategies may also be of interest. In experimental models, restoration of short-chain fatty acids or transfer of a healthier microbial community has been associated with improved epithelial barrier integrity and reduced inflammatory signaling [53,55].

Collectively, KHK, PKM2, and AKR1B1 represent pharmacologically tractable components of fructose-related metabolism with supportive preclinical evidence in CRC and varying degrees of clinical experience in other diseases. However, none of these approaches have been clinically validated as a fructose-targeted therapy in CRC. Whether these pathways represent actionable vulnerabilities in CRC and whether they have particular relevance to eoCRC remain to be determined.

## 8. Current American Dietary Guidelines Recommendations on Added Sugar Intake

Given the established associations between high added-sugar intake and adverse metabolic health outcomes, major health organizations recommend limiting added sugars as part of an overall healthy dietary pattern. Quantifying individual dietary fructose intake remains challenging because the U.S. Food and Drug Administration (FDA) Nutrition Facts labels report total and added sugars rather than individual monosaccharides. Consequently, fructose derived from added sweeteners is incorporated within the broader category of added sugars, which may include sucrose, HFCS, syrups, honey, and fruit juice concentrates. Current dietary recommendations therefore focus primarily on limiting added sugars and SSBs and emphasizing nutrient-dense dietary patterns rather than establishing fructose-specific intake thresholds.

The American Heart Association (AHA) also recommends choosing minimally processed foods instead of ultra-processed foods. However, ultra-processed foods terminology and classification remain variable because no universally accepted classification system exists. The commonly used NOVA system classifies UPFs primarily according to the extent and purpose of industrial processing and the presence of ingredients or additives not typically used in home cooking, rather than nutrient composition alone [67]. Current recommendations relevant to added sugars and overall dietary patterns are summarized in Table 3.

## 9. Conclusions

Emerging epidemiologic and experimental evidence supports a potential role for excessive fructose and sugar-sweetened beverage consumption in colorectal carcinogenesis, including possible relevance to early-onset colorectal cancer. Fructose may promote tumor development through metabolic reprogramming, altered lipid synthesis, hypoxic adaptation, and stromal and immune–metabolic interactions, as well as disruption of the intestinal barrier and gut microbiome. However, current human epidemiologic and clinical evidence does not establish dietary fructose as a causative agent in colorectal carcinogenesis. Direct evidence linking these mechanisms specifically to human eoCRC remains limited, and the effects of fructose appear to depend on dose, food matrix, energy balance, and host–microbiome context.

Future research should focus on feasible approaches that strengthen the connection between dietary exposure and colorectal neoplasia, including prospective observational studies with improved dietary assessment, studies relating fructose-associated microbial or metabolic features to colorectal precursor lesions or tumor characteristics, and comparative molecular analyses of early-onset and average-onset CRC. Because long-term controlled dietary studies with CRC incidence as an endpoint are impractical, observational and translational studies will remain important but should carefully address residual confounding and distinguish refined added sugars and SSBs from whole-food sources of fructose. From a public health perspective, current evidence does not support fructose-specific cancer prevention recommendations beyond existing guidance to limit added sugars and sugar-sweetened beverages.

## Figures and Tables

**Table 1:** Alterations in fructose-related metabolic pathways and their functional consequences in colorectal cancer.

Pathway	Altered Component	Mechanistic Effects	Impact on Cancer Cells	References
Fructose transport	GLUT5 upregulation	Increases cellular fructose uptake and utilization in CRC cells and cancer-associated fibroblasts	Promotes tumor growth, chemotherapy resistance and metastasis	[33,34,35,36]
Fructose phosphorylation	KHK-A activation	Promotes phosphorylation and nuclear translocation of PKM2, linking fructose metabolism to transcriptional and signaling programs	Drives EMT and liver metastasis	[37]
Triose cleavage	ALDOB overexpression	Enhances cleavage of F1P into triose intermediates and supports glycolytic metabolic reprogramming, particularly under hypoxic conditions	Enhances tumor survival in hypoxic niches and metastatic colonization	[38,39]
Polyol pathway initiation	AKR1B1 overexpression	Increases glucose-to-sorbitol conversion, promotes reactive oxygen species generation and mitogenic signaling, and influences M2 TAM polarization	Promotes G1/S cell-cycle progression, EMT, and a tumor-supportive immune microenvironment	[40,41,42]
Polyol redox cycling and mevalonate pathway	SORD-mediated reverse flux	Glucose–fructose co-exposure promotes reverse SORD flux from fructose to sorbitol, alters the NAD+/NADH ratio, and activates the mevalonate pathway	Enhances tumor cell motility and metastatic potential	[43]
Hypoxic metabolic adaptation	Necroptosis pathway inhibition	Fructose sustains glycolytic flux and ATP production under hypoxic stress and suppresses RIP-dependent necroptotic signaling	Preserves CRC-cell viability under hypoxia	[44]
Immune-metabolic regulation	HK2 activation	High-fructose exposure enhances HK2-dependent glycolysis and impairs M1-like TAM polarization	Supports tumor-promoting immune microenvironment and CRC progression	[45,46]
Angiogenic signaling	ROS/Akt/Src–VEGF axis activation	Increases ROS and activates Akt/Src signaling, resulting in increased VEGF expression	Promotes tumor angiogenesis and increases microvascular density	[47]

**Table 2:** Effect of high-fructose dietary interventions on gut microbiota composition and host physiology in human studies.

Study & Design	Fructose Exposure	Key Microbial Findings	Functional, Metabolic, and Inflammatory Impacts
Beisner et al., 2020 [10] Pilot open-label single-arm study; N = 12 healthy women	Four consecutive 1-week phases: Week 1: Low-fructose diet, 10 g/day; Week 2: Fruit & and vegetable diet, 100 g/day; Week 3: low-fructose diet, 10 g/day; Week 4: high-fructose syrup (HFS), 100 g/day)	Fruit and vegetable diet • ↑Firmicutes, ↓Bacteroidetes • ↑Butyrate producers (*Faecalibacterium*, *Anaerostipes*, Erysipelatoclostridium) HFS diet ↓Firmicutes, ↑Bacteroidetes ↓Butyrate producers *Faecalibacterium* Erysipelatoclostridium ↓Ruminococcus	Bacteroidetes abundance positively correlated with plasma total cholesterol and LDL, whereas Firmicutes showed an inverse correlation. HFS exposure was associated with altered inositol phosphate-related pathways.
Aleman et al., 2021 [63] Pilot double-blind crossover randomized controlled trial; N = 10 participants with BMI 30–40 kg/m^2^	75 g/day pure fructose (~20% of daily energy intake) vs. isocaloric glucose for 14 days, separated by a 19-day washout	↓*Bifidobacterium* relative abundance in both fructose and glucose arms No significant change in overall microbial diversity	• ↑Fecal fructose • No significant change in intestinal permeability, endotoxemia markers (LBP, sCD14), or gastrointestinal inflammatory markers (fecal calprotectin, I-FABP).

↑ = increased relative abundance; ↓ = decreased relative abundance.

**Table 3:** Current Dietary Recommendations Relevant to Added Sugar Intake.

Dietary Aspect	AHA 2026 Dietary Guidance Health [68,69,70]	Dietary Guideline for Americans 2025–2030 [71]
Added sugar restriction	Minimizing intake of added sugars in beverages and foods: • Men: <36 g per day (150 calories or 9 teaspoons) • Women and children: <25 g per day (100 calories or 6 teaspoons)	Avoid SSBs and foods with added sugar • Limit added sugars to <10 g per meal
Whole fruits & vegetables	Eating plenty of vegetables and fruits and choosing a wide variety	Eat 3 servings of vegetables and 2 servings of fruits per day in whole, nutrient-dense form
Whole grains & fiber	Choosing foods made mostly with whole grains rather than refined grains	Prioritize whole grains (2–4 servings/day); limit refined carbohydrates (white bread, packaged cereals)
Processed foods	Choosing minimally processed foods instead of ultra-processed foods	Avoid highly processed packaged foods; prioritize whole foods to support gut microbiome diversity

## Data Availability

No new data were created or analyzed in this study. Data sharing is not applicable to this article.
